# A comprehensive 10-year review of decannulation and perioperative complications in pediatric open airway reconstruction

**DOI:** 10.1007/s00405-026-10154-7

**Published:** 2026-04-23

**Authors:** Hen Chaushu, Alexandra Dorman, Oshri Wasserzug, Ari DeRowe, Orna Katz

**Affiliations:** 1 Department of Otolaryngology-Head and Neck Surgery and Maxillofacial Surgery, Tel Aviv Sourasky Medical Center, 6 Weizmann Street, Tel Aviv, 6423906 Israel; 2 Affiliated to Gray Faculty of Medical & Health Science, Tel-Aviv University, Tel Aviv, Israel

**Keywords:** Laryngotracheal Stenosis, Laryngoplasty, Airway Management, Reconstruction Surgical Procedures, Postoperative Complications

## Abstract

**Purpose:**

To characterize perioperative morbidity, microbiologic findings and decannulation outcomes in pediatric open airway reconstruction.

**Methods:**

All children undergoing open airway reconstruction between 2014 and 2024 were included. Demographic characteristics, stenosis grade, surgical technique, staging strategy, stent use, graft configuration, postoperative complications, microbiological findings, and decannulation outcomes were analyzed.

**Results:**

Forty-four children (mean age, 4.1 ± 2.9 years) underwent open airway reconstruction, most with high-grade stenosis (60% grade III, 34% grade IV). Procedures included laryngotracheoplasty (*n* = 35), cricotracheal resection (*n* = 7), and tracheal resection (*n* = 2). Single-stage reconstruction was performed in 24 patients (54.5%) and double-stage in 20 (45.5%). Airway stents were used in 31 of 44 patients (70.5%), with a significant decline over time (85.7% vs. 43.8%; *P* < .01). Overall, 70.5% achieved decannulation, including 68.2% within 6 months. Single-stage reconstruction was associated with a shorter median time to decannulation compared with double-stage reconstruction (median, 0.26 vs. 3.27 months; *P* = .004). All postoperative infections occurred in stented patients (29% vs. 0%; *P* = .03(, representing a more clinically complex subgroup. Multivariable analysis demonstrated an independent association between stenosis severity and delayed decannulation.

**Conclusion:**

Outcomes after pediatric open airway reconstruction were associated with surgical staging, stent use, and perioperative factors. These findings support individualized treatment strategies and outcome assessment beyond decannulation alone.

**Supplementary Information:**

The online version contains supplementary material available at 10.1007/s00405-026-10154-7.

## Introduction

Pediatric laryngotracheal stenosis (LTS) remains a significant source of airway related morbidity and a common indication for open airway reconstruction in children [1–2]. Although advances in neonatal care and endoscopic techniques have reduced open airway approach incidence, many children still require open surgical intervention to achieve airway patency, voice preservation, and decannulation [3]. These reconstructions, most commonly laryngotracheoplasty (LTP) or cricotracheal resection (CTR), are complex, resource-demanding, and require long-term multidisciplinary management [4–5].

While decannulation is often the primary surgical endpoint, it does not capture the full burden of perioperative complications, nor the diverse factors influencing patient outcomes [6]. Postoperative challenges such as granulation tissue, infection, stent-related morbidity, withdrawal syndrome, and the need for revision procedures remain prevalent, particularly in children with comorbidities, multilevel stenosis, or prolonged tracheostomy dependence [7]. Understanding these complications is essential, especially as perioperative care protocols and stent use strategies continue to evolve.

Recent literature has provided insights into surgical techniques. Meta-analyses and multicenter reviews have shown that single stage (SS) LTP is associated with higher early decannulation rates compared to double-stage (DS) procedures, particularly in patients with isolated subglottic disease or favorable anatomy [8–9]. However, when controlling stenosis grade, this advantage narrows. DS LTP may still be preferred in patients with glottic involvement, significant comorbidities, or poor tolerance of prolonged intubation [10]. Meanwhile, the use of intraluminal stents remains controversial. Some series show improved airway stability with prolonged stent use [11], while others associate stents with increased rates of airway infection, biofilm formation and granulation tissue requiring debridement [12].

Beyond technical choices, variability in sedation techniques, intensive care unit (ICU) protocols and perioperative antibiotic treatment protocols contribute to heterogeneity in complication rates. For instance, airway cultures often reveal colonization by *S. aureus*, *P. aeruginosa*, and *Candida* spp., which may exacerbate graft healing complications and guide perioperative antibiotic selection [10]. Similarly, prolonged sedation after SS repair, although essential to graft healing, can increase the risk of iatrogenic withdrawal syndrome, necessitating structured weaning protocols and multidisciplinary ICU care.

Despite growing literature on operative techniques, few contemporary studies have comprehensively evaluated decannulation, perioperative morbidity, microbiologic findings, and evolving stent practices within a single pediatric airway reconstruction cohort. This study presents a 10-year retrospective experience aimed at characterizing surgical outcomes, complication patterns, and predictors of decannulation in children undergoing open airway reconstruction.

## Methods

### Study design and setting

We conducted a retrospective cohort study of pediatric patients who underwent open airway reconstruction at tertiary airway referral center, between January 2014 and August 2024. The study was approved by the Institutional Review Board and performed in accordance with the Declaration of Helsinki.

### Patient selection

Eligible patients were children aged 0–18 years who underwent open airway reconstruction for laryngotracheal stenosis or related airway pathology. Patients treated exclusively with endoscopic dilation procedures, those undergoing repair of laryngeal clefts without stenosis, and those with incomplete medical records were excluded. Clinical, radiologic, and operative records were reviewed for all patients.

### Preoperative evaluation

All patients underwent standardized preoperative assessment, including direct laryngotracheal endoscopy under general anesthesia, with grading of stenosis according to the Cotton–Myer [13] classification. Radiographic imaging (CT or MRI) was obtained when 3D modeling was considered [14] and when complete obstruction of the airway (grade IV) was found on DL. Demographic characteristics, comorbidities, prior airway interventions, and tracheostomy status were extracted from electronic medical records.

### Surgical technique

Open airway reconstruction was performed using one of the following techniques: LTP, CTR or tracheal resection (TR). Surgical approach was determined based on stenosis severity, length, and anatomical location. Autologous costal cartilage or thyroid ala grafts were used for LTP as appropriate. Intraoperative variables included the surgical approach (open vs. combined endoscopic–open), staging strategy (SS vs. DS reconstruction), graft configuration (anterior, posterior, or combined), duration of surgery, and intraoperative complications. Intraoperative tracheostomy-site cultures were obtained at the beginning of each reconstruction procedure, prior to airway manipulation.

**Fig. 1:**
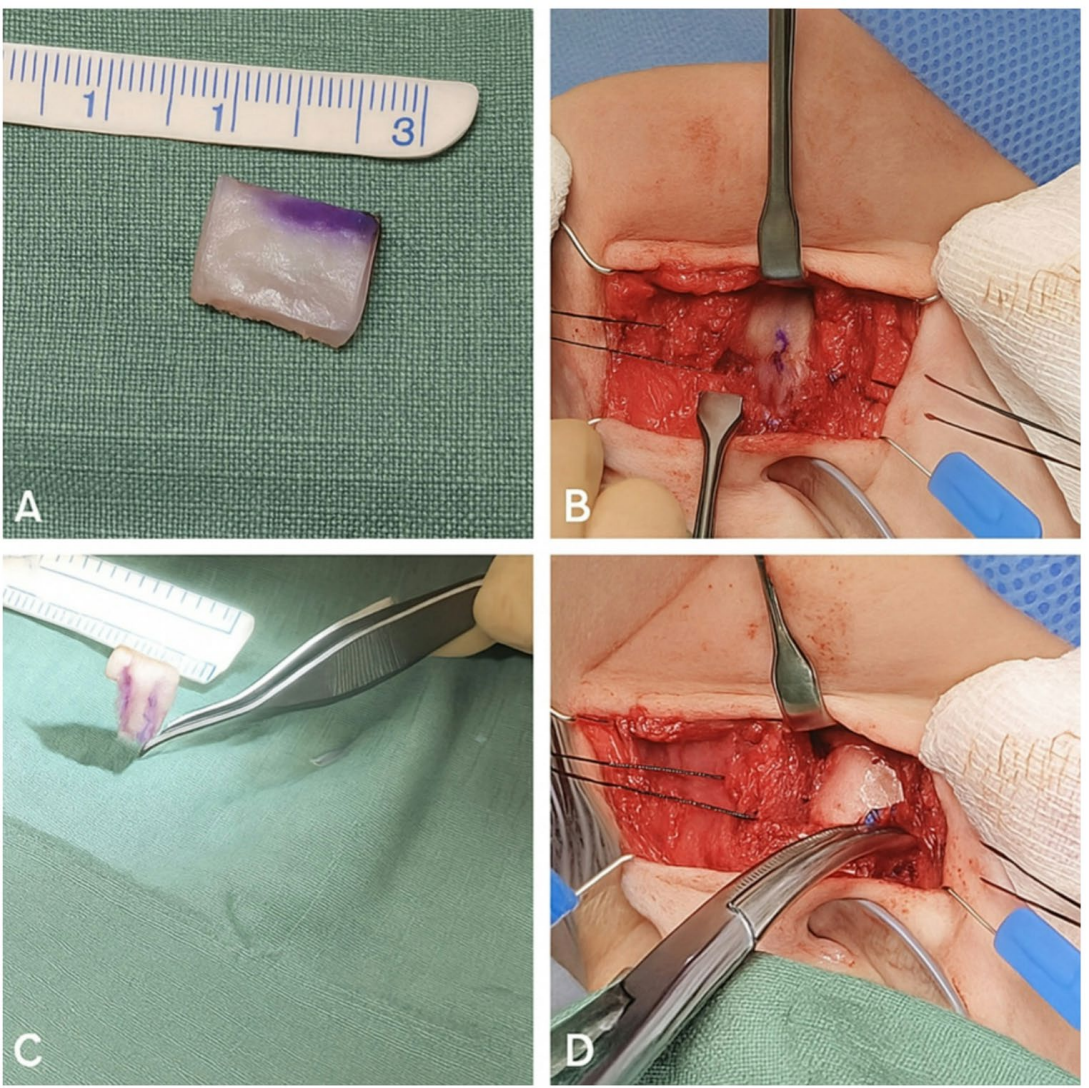
Intraoperative Images Demonstrating Graft Preparation and Implantation during Double Staged Laryngotracheal Reconstruction. (**A**) Autologous costal cartilage graft harvested. (**B**) Operative field exposure following midline laryngofissure; graft site prepared with sutures in place for stabilization. (**C**) Graft shaped for anterior augmetion. (**D**) Cartilage graft inset into the anterior airway defect and secured in position with fine sutures under tension-free closure

Per institutional protocol, all patients received empiric piperacillin- tazobactam, which was later tailored based on microbiological results. Representative intraoperative steps of graft harvesting and placement are shown in Fig. 1. The decision to use airway stent was made by the operating surgeon based on intraoperative assessment of airway stability, stenosis severity, and reconstruction complexity.

The decision to perform SS versus DS reconstruction was made by the operating surgeon based on airway severity, anatomical complexity, tracheostomy involvement, and overall patient stability. SS reconstruction was generally reserved for neurologically stable children able to tolerate prolonged postoperative intubation and sedation. DS reconstruction was favored in patients with multilevel disease, higher-grade stenosis, significant medical comorbidities (e.g., pulmonary or cardiac disease), or when prolonged airway protection via tracheostomy was deemed necessary.

### Postoperative management and data collection

Postoperative variables included local infection (including graft infection, neck infection and dehiscence) pulmonary complications (pneumothorax, pneumonia), duration of stent placement (if applicable), length of stay, time to decannulation, the need for revision surgery and follow up length.

Postoperative infection was defined as the presence of clinical signs of surgical site infection requiring systemic antibiotic therapy. Clinical criteria included one or more of the following: erythema, purulent drainage, graft instability, wound dehiscence with inflammatory signs, fever with localizing findings, or documented abscess formation. Radiographic confirmation (when obtained) and microbiologic growth supported the diagnosis but were not required in isolation. Diagnosis was established by the attending pediatric otolaryngology team in conjunction with the treating department when applicable. Positive microbiologic culture in the absence of clinical or radiographic signs of infection was considered colonization and was not classified as postoperative infection.

Per institutional protocol, SS reconstruction involves planned postoperative intubation with continuous sedation for graft protection, typically lasting 3–5 days. In contrast, DS reconstruction maintains tracheostomy patency postoperatively and does not routinely require prolonged intubation or deep sedation.

### Outcomes

The primary outcome was successful decannulation (yes/no). Secondary outcome measures included time to decannulation and hospital length of stay. Postoperative adverse events were analyzed separately as complications, including surgical site infection, pneumonia, graft-related complications, and withdrawal syndrome.

### Statistical analysis

All analyses were performed using IBM SPSS Statistics for Windows, Version 25.0 (IBM Corp., Armonk, NY, USA). Categorical variables were summarized as frequencies and percentages. Continuous variables were assessed for distribution using histograms; normally distributed variables were reported as mean ± standard deviation (SD), and non-normally distributed variables as median with interquartile range (IQR).

Categorical variables were compared using Fisher’s exact test. Continuous variables were compared using the independent-samples t-test or Mann–Whitney U test, as appropriate. Associations between decannulation status and categorical predictors were assessed using the log-rank test, while associations with continuous variables were explored using univariate Cox regression analysis. All tests were two-sided, and a p-value < 0.05 was considered statistically significant.

Median time to decannulation between groups was compared using the Mann–Whitney U test due to non-normal distribution. Time-to-event distributions were analyzed using Kaplan–Meier survival curves and compared using the log-rank test.

Multivariable logistic regression was used to identify independent predictors of successful decannulation and postoperative infection. Multivariable Cox proportional hazards regression was used to identify independent predictors of time to decannulation. Variables included in the multivariable model were selected based on clinical relevance and univariate analysis with *P* < .10.

## Results

**Fig. 2:**
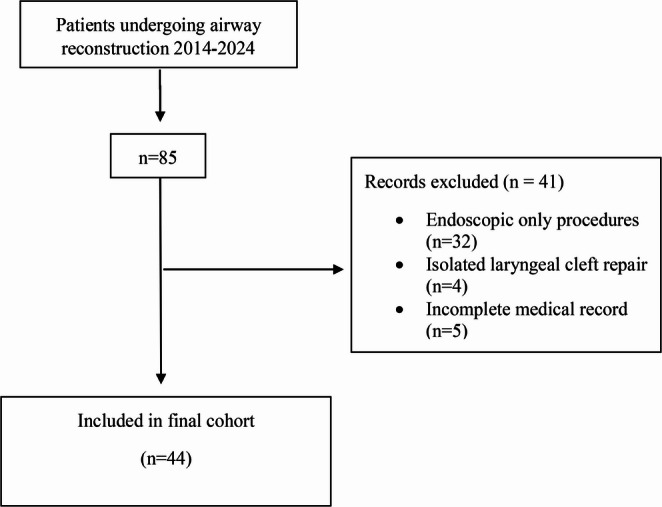
Intraoperative graft placement. (**A**) The laryngotracheal defect after midline incision and expansion of the airway framework. (**B**) Placement of the cartilage graft into the defect, positioned to widen the airway and restore the laryngotracheal contour.

A total of 85 patients underwent open airway reconstruction between 2014 and 2024. Forty-one were excluded (32 endoscopic-only procedures, 4 isolated laryngeal cleft repairs without stenosis, and 5 incomplete medical records), resulting in 44 patients included in the final cohort (Fig. 2). The mean age at surgery was 4.1 ± 2.9 years, and 40.9% were female. Prematurity was present in one-third of patients with available data, and comorbidities were frequent, including neurological (38.6%), cardiac (29.5%), craniofacial anomalies (22.7%) and pulmonary disease (18.2%). Among 35 children with documented grading, most had high-grade subglottic stenosis (60.0%–Cotton-Myer grade III and 34.3% grade IV). These baseline characteristics are summarized in Table 1.

**Table 1:** Baseline Demographic and Clinical Characteristics (*n* = 44)

Characteristic	Value
Age at surgery, years, median (IQR)	3.2 (1.9–5.4)
Female sex	18/44 (40.9%)
Preterm birth	10/30 (33.3%)
Comorbidities	
Neurological	17/44 (38.6%)
Cardiac	13/44 (29.5%)
Craniofacial	10/44 (22.7%)
Pulmonary	8/44 (18.2%)
Down syndrome	2/44 (4.5%)
Grade of stenosis	
II	2/35 (5.7%)
III	21/35 (60.0%)
IV	12/35 (34.3%)

*SD* standard deviation, *IQR* interquartile range

*Preterm birth data was available for 30 patients

Stenosis grade documented in 35 patients

The cohort was predominantly treated with open surgical approaches: 36 of 44 children (81.8%) underwent open airway reconstruction, while 8 (18.2%) underwent combined endoscopic airway surgery with open graft harvest. LTP was performed in 35 patients (79.5%), CTR in 7 (15.9%) and segmental tracheal resection in 2 (4.5%). Twenty-four children (54.5%) were managed with SS reconstruction and 20 (45.5%) with a DS reconstruction. A stent was used in 31 patients (70.5%), most commonly an endotracheal tube, whereas 13 (29.5%) were managed without a stent. Stent use decreased substantially over the study period. During the first 5 years, 24 of 28 children (85.7%) were managed with a stent compared with 7 of 16 (43.8%) in the subsequent 5 years (*P* < .01).

**Fig. 3:**
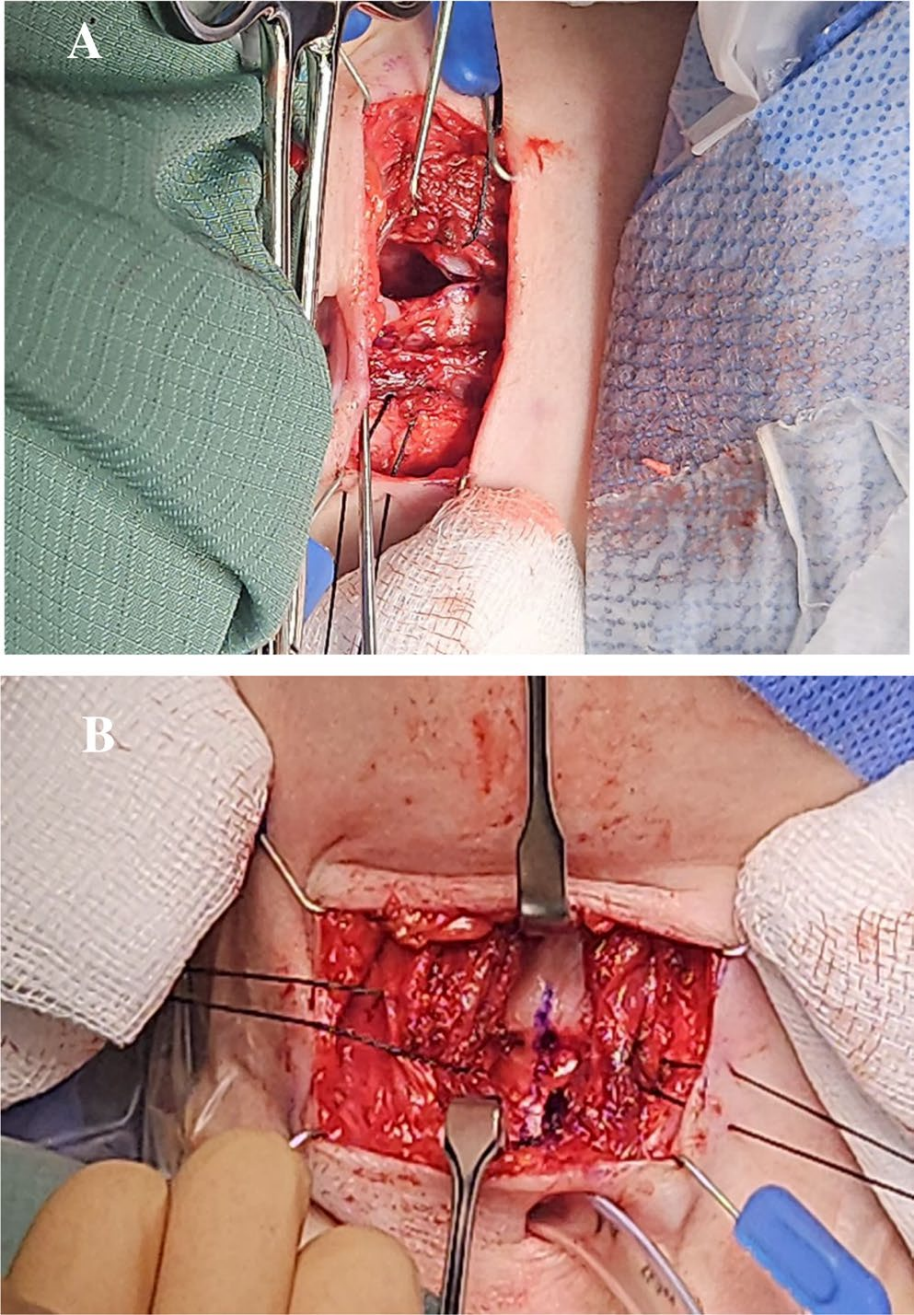
Intraoperative graft placement

Cartilage grafting patterns differed significantly between SS and DS procedures. In SS cases, reconstruction was typically performed with an anterior graft alone (16/24, 66.7%), and no posterior grafts were used. In DS cases, grafting was more complex and often posteriorly based: 9/20 (45.0%) had anterior-only grafts, 8/20 (40.0%) posterior grafts, and 3/20 (15.0%) no graft (*P* = .003 for graft distribution between SS and DS). Detailed surgical characteristics are presented in Table 2 and examples of anterior graft positioning are demonstrated in Fig. 3.

**Table 2:** Surgical characteristics and decannulation outcomes

Variable / Outcome	Value
Open surgery	36/44 (81.8%)
Combined endoscopic surgery	8/44 (18.2%)
Laryngotracheoplasty	35/44 (79.5%)
Cricotracheal resection	7/44 (15.9%)
Tracheal resection	2/44 (4.5%)
Single-stage reconstruction	24/44 (54.5%)
Double-stage reconstruction	20/44 (45.5%)
Anterior graft only	25/44 (56.8%)
Posterior graft only	9/44 (20.5%)
Anterior and posterior graft	2/44 (5.0%)
Stent used	31/44 (70.5%)
Successful decannulation (any time)	31/44 (70.5%)
Follow-up time, days, median (IQR)	159 (89–881)
Tracheostomy at last follow-up	7/44 (15.9%)

IQR – interquartile range

### Infectious and pulmonary complications

Postoperative surgical site infection, as defined by clinical criteria requiring systemic antibiotic treatment, occurred in 9 children (20.5%). These included graft infection, neck infection, and clinically significant wound dehiscence. Univariate analysis identified several factors associated with postoperative infection (Table 3).

**Table 3:** Predictors of postoperative infection

Variable	No infection (*n*=35)	Infection (*n*=9)	*P*-value
Age, median (IQR), years	3.25 (2.35–5.40)	2.43 (1.74–3.58)	0.302
Male sex, n (%)	15 (42.9%)	3 (33.3%)	0.716
Duration of tracheostomy prior to surgery, median (IQR), months	29.00 (21.78–46.17)	18.90 (16.83–20.87)	**0.042**
Single-stage reconstruction, n (%)	18 (51.4%)	6 (66.7%)	0.477
Stent use, n (%)	22 (62.9%)	9 (100.0%)	**0.041**
Duration of stent placement, median (IQR), days	6.00 (0.00–8.00)	7.00 (6.00–7.00)	0.248
Length of hospital stay, median (IQR), days	11.00 (9.00–19.00)	19.00 (12.00–22.00)	0.085

†Mann–Whitney U test for continuous variables; Fisher exact test for categorical variables

All infections occurred in patients who required airway stent (100% vs. 62.9%, *P* = .041). Patients requiring stents generally had more severe or anatomically complex airway disease.

In addition, patients who developed postoperative infection had a significantly shorter duration of tracheostomy prior to reconstruction (median 18.9 vs. 29.0 months, *P* = .042). Length of hospital stay showed a trend toward prolongation in infected patients, although this did not reach statistical significance (*P* = .085).

Pulmonary complications including pneumonia and pneumothorax were documented in 6 children (13.6%) without stage- or stent-specific differences (*P* > .20 for all comparisons). Revision airway surgery was required in 9 children (20.5%), including 4 SS patients (16.7%) and 5 DS patients (25.0%) (*P* = .76).

### Withdrawal syndrome

Withdrawal syndrome, reflecting medication-related withdrawal following prolonged postoperative sedation and analgesia, occurred in 15 of 44 patients (34.1%). Withdrawal syndrome occurred more frequently in the SS group compared with the DS group (50.0% vs. 15.0%, *P* = .02).

### Microbiology

Intraoperative tracheostomy-site cultures were obtained at the beginning of each procedure and primarily reflect airway colonization patterns rather than postoperative infection. Culture results were used to tailor perioperative antibiotic therapy but were not, in isolation, considered diagnostic of infection.

Bacterial cultures obtained intraoperatively were positive in 31 of 44 children (70.5%), reflecting baseline airway colonization. Pseudomonas aeruginosa was the most common isolate (17/44, 38.6%), followed by Staphylococcal species (8/44, 18.2%) and other organisms (12/44, 27.3%). Colonization patterns did not differ significantly between SS and DS reconstruction, except for a higher frequency of non-Pseudomonas/Staphylococcal organisms in the SS group (41.7% vs. 10.0%, *P* = .04). These microbiologic findings were used to tailor perioperative antibiotic therapy but were not, in isolation, considered evidence of infection.

The high prevalence of positive cultures in this cohort likely reflects chronic tracheostomy-associated colonization rather than active infection.

### Decannulation outcomes

**Fig. 4:**
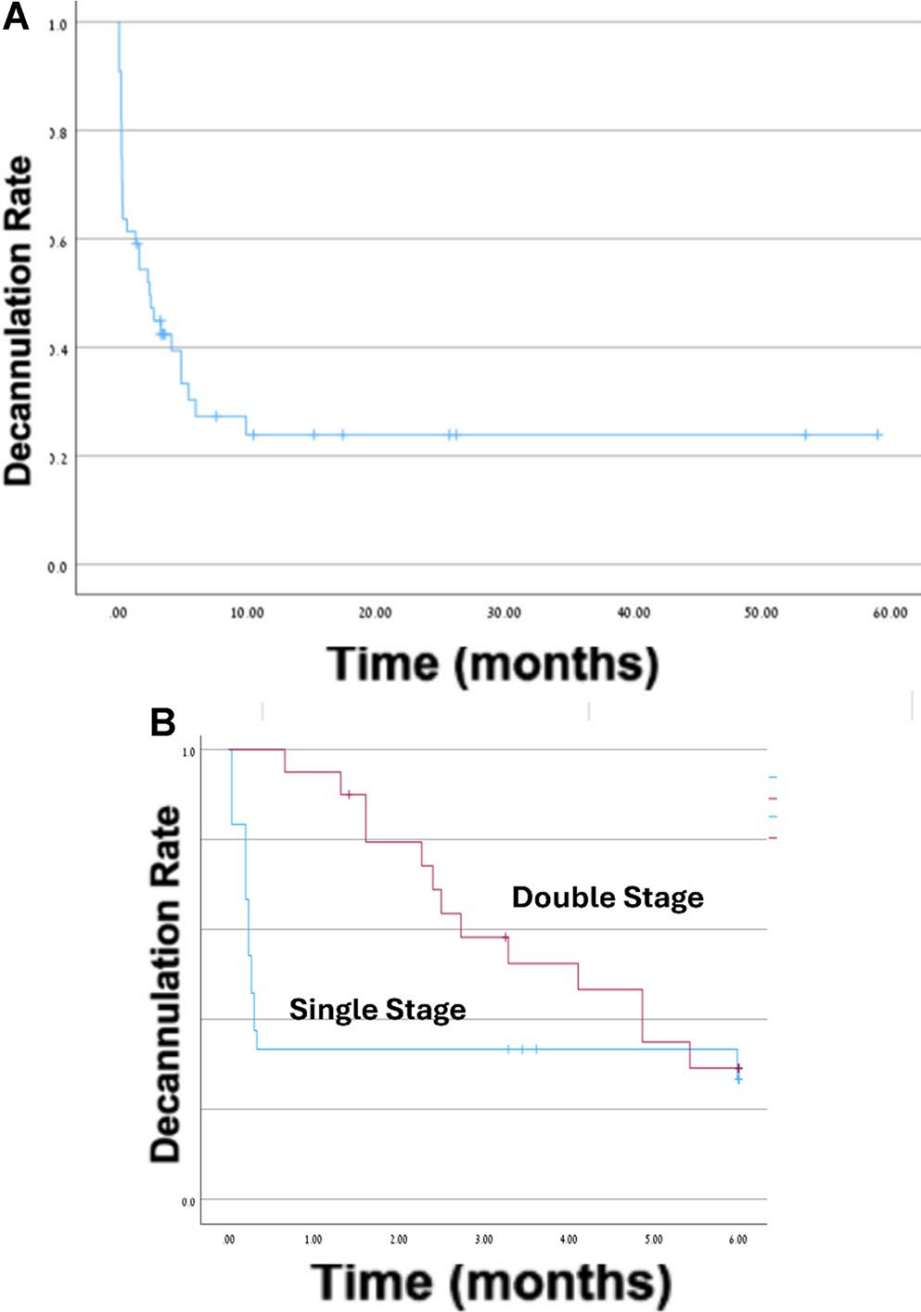
Kaplan–Meier curve for time to decannulation following airway reconstruction

Overall, 31 of 44 patients (70.5%) achieved successful decannulation during follow-up, and 30 patients (68.2%) were successfully decannulated within 6 months of surgery, as shown in Fig. 4a, representing the primary endpoint for the cohort. Kaplan–Meier analysis did not demonstrate a significant difference in time-to-decannulation across staging strategies, stent use status, graft type, or surgical technique (log-rank *P* > .05 for all comparisons). Although median time to decannulation differed significantly between groups, Kaplan–Meier analysis did not demonstrate a statistically significant difference in the overall time-to-event distribution. This discrepancy likely reflects early clustering of decannulation events in the SS group, with convergence of curves over longer follow-up. The median number of endoscopic assessments (direct laryngoscopy/bronchoscopy) prior to decannulation was 2.2 procedures.

Despite similar ultimate decannulation rates, the time of decannulation differed between groups. Children who were decannulated within 6 months exhibited a particularly rapid trajectory, with SS patients decannulating at a median of 0.23 months compared with 2.50 months in DS patients), depicted in Fig. 4b. Stent use did not influence decannulation success (71.0% vs. 69.2%, *P* = 1.00), nor time to decannulation (*P* = .21). Given the non-randomized design, these associations should be interpreted as reflective of underlying case selection rather than causal effects of surgical staging. In a sensitivity analysis restricted to patients with ≥ 6 months of follow-up (*n* = 29), the overall decannulation rate was 89.7%. Ultimate decannulation success did not differ significantly between SS and DS reconstruction (93.3% vs. 85.7%, *P* > .05).

Revision airway surgery was required in 9 children (20.5%), including 4 SS patients (16.7%) and 5 DS patients (25.0%) (*P* = .76).

Median follow-up was 159 days (interquartile range [IQR], 89–881), with a wide range up to more than 9 years. At last follow-up, 7 of 44 children (15.9%) remained tracheostomy dependent.

Univariate analysis was performed to identify factors associated with successful decannulation and time to decannulation (Tables 4 and 5). Stenosis severity was significantly associated with decannulation outcomes. Children with higher-grade stenosis were less likely to achieve successful decannulation, with grade IV stenosis overrepresented among patients who remained tracheostomy dependent (53.8% vs. 16.1%, *P* = .017; Table 4).

**Table 4:** Predictors of successful decannulation

Variable	No Decannulation (*n*=13)	Decannulation (*n*=31)	*P*-value
Age, median (IQR), years	4.51 (2.45–7.29)	2.97 (1.97–4.35)	0.173
Male sex, n (%)	4 (30.8%)	14 (45.2%)	0.507
Duration of tracheostomy prior to surgery, median (IQR), months	49.77 (21.57–61.90)	25.03 (17.92–38.30)	0.064
Preoperative weight, median (IQR), kg	17.00 (10.85–21.25)	13.45 (10.55–16.25)	0.231
Pulmonary comorbidity, n (%)	3 (23.1%)	5 (16.1%)	0.676
Cardiac comorbidity, n (%)	6 (46.2%)	7 (22.6%)	0.155
Down syndrome, n (%)	2 (15.4%)	0 (0.0%)	0.082
Stent use, n (%)	9 (69.2%)	22 (71.0%)	>0.999
Local infection, n (%)	3 (23.1%)	6 (19.4%)	>0.999
Pulmonary complication after surgery, n (%)	3 (23.1%)	3 (9.7%)	0.339
Grade of stenosis			
II	0/13 (0%)	2/31 (6.5%)	
III	3/13 (23.1%)	18/31 (58.1%)	
IV	7/13 (53.8%)	5/31 (16.1%)	0.017
Suprastomal Granulation after Surgery, n (%)	5 (38.5%)	15 (48.4%)	0.724

‡ P-values derived from Fisher exact test

† Stenosis grade available in 35 patients; percentages calculated accordingly

**Table 5:** Univariate cox proportional hazards regression analysis of key predictors of time to decannulation

Variable	Hazard Ratio (HR)	95% CI	*P*-value
Age at surgery, per year	0.92	0.80–1.07	0.278
Male sex	1.07	0.54–2.13	0.849
Cotton–Myer stenosis grade IV (vs. grade II–III)	0.20	0.04–0.95	**0.042**
Duration of tracheostomy prior to surgery, per month	0.99	0.97–1.01	0.165
Stent use	1.25	0.59–2.65	0.566
Number of endoscopic procedures prior to decannulation, per procedure	0.29	0.18–0.46	**< 0.001**
HR > 1 indicates shorter time to decannulation			

HR < 1 indicates prolonged time to decannulation

In time-to-event analysis using Cox proportional hazards regression (Table 5), higher stenosis severity was independently associated with delayed decannulation: Cotton–Myer grade IV stenosis demonstrated a significantly lower likelihood of early decannulation compared with grade II–III stenosis (HR 0.20, 95% CI 0.04–0.95, *P* = .042). Additionally, a greater number of endoscopic airway procedures prior to decannulation was strongly associated with delayed decannulation, as expected (HR 0.29 per procedure, 95% CI 0.18–0.46, *P* < .001).

Multivariable logistic regression analysis demonstrated that stenosis severity was independently associated with successful decannulation (OR 0.08, 95% CI 0.01–0.69, *P* = .021). Age, surgical technique, and stent use were not independently associated with decannulation success.

Multivariable analysis of postoperative infection did not identify independent predictors, likely reflecting the limited number of infection events and the strong association between infection and stent use observed in univariate analysis. These findings indicate that higher stenosis severity demonstrated an independent statistical association with delayed decannulation, while operative technique and stent use were not independently associated with decannulation outcomes.

## Discussion

Open airway reconstruction in children requires individualized decision-making, balancing disease severity, comorbidities, airway anatomy, and the child’s ability to tolerate postoperative care. Contemporary literature consistently emphasizes tailoring the operative technique to patient and disease-specific factors rather than adopting a uniform approach for all children with subglottic or tracheal stenosis [15].

### Choosing single-stage vs. double-stage reconstruction

The fundamental principle guiding SS reconstruction is the desire to avoid a long-term tracheostomy. SS is therefore reserved for children who can tolerate several days of postoperative intubation and sedation, typically those who are neurologically intact, behaviorally cooperative, and without significant pulmonary fragility [9, 16]. In addition, when the stoma contributes to the stenosis or is itself part of the pathologic segment, SS provides the advantage of simultaneous airway expansion and definitive stoma closure [17].

Conversely, DS reconstruction is favored in more complex cases. This includes children with multilevel disease, high-grade stenosis, severe or poorly controlled comorbidities (pulmonary, cardiac, or neurologic), or a history of failed prior reconstruction. DS procedures confer several advantages in these scenarios: the child avoids the physiological stress of early extubation, the airway is protected by the tracheostomy, and the surgeon retains a controlled airway via the tracheostomy while the reconstruction heals [16–17]. DS reconstruction is also preferred when the stoma is minimally involved or not part of the disease process, since its presence can safely support the postoperative period. Revision cases, especially failed LTP, are likewise more commonly approached with DS techniques to reduce the risk of restenosis and allow closer airway surveillance [18].

Our cohort supports these principles. SS patients demonstrated substantially faster decannulation than DS patients (median 0.26 vs. 3.27 months). These differences likely reflect underlying differences in disease severity and perioperative management inherent to staging selection. The distribution of graft types also reflected this logic: SS cases predominantly used anterior grafts, whereas DS cases more often required posterior grafting, reflecting greater anatomic complexity.

Importantly, surgical staging and stent utilization were not randomly assigned but selected according to airway severity, anatomic complexity, and patient comorbidities. SS reconstruction was generally reserved for neurologically stable children able to tolerate prolonged postoperative intubation, whereas DS procedures were more commonly performed in patients with multilevel disease, higher-grade stenosis, or significant comorbidity. Accordingly, observed differences in time to decannulation, infection rates, and withdrawal syndrome likely reflect differences in underlying disease complexity and perioperative management rather than intrinsic effects of staging strategy or stent placement.

### Considerations for stent use

Airway stents are considered to play an important role in LTP [19–20] although their role has evolved considerably. While they were once placed routinely to provide postoperative graft support, accumulating experience has highlighted their potential drawbacks, including disruption of airway clearance, rapid microbial and subsequent inflammatory or obstructive sequelae [21–22]. These concerns have pushed practice toward a more restrained approach, using stents only when structural reinforcement is essential and limiting their duration whenever possible [23]. This changing philosophy was clearly reflected in our data: stent placement, which characterized 86% of procedures in the earlier study period, fell to 44% in the later years (*P* < .01), underscoring a growing preference for stent-sparing techniques as confidence with graft stability and postoperative management has increased.

Similarly, stent utilization was heavily confounded by airway severity and reconstruction complexity. Stents were preferentially used in more severe or unstable reconstructions. Therefore, the association between stent use and postoperative infection should not be interpreted as evidence of causation, but rather as a marker of increased baseline airway complexity.

The marked decline in stent utilization over the study period likely reflects evolving surgical philosophy and increasing confidence with graft stability rather than changes in disease severity alone. However, temporal changes introduce additional heterogeneity and potential confounding.

### Decannulation outcomes

Our cohort demonstrated a 71% decannulation rate within 6 months, consistent with reported early decannulation rates of 60–85% following modern open reconstruction techniques [24]. As in other series, ultimate decannulation is expected to exceed early rates, often surpassing 85–90% in long-term follow-up.

Importantly, we found no significant difference in decannulation probability between SS and DS approaches, aligning with meta-analytic data showing equivalent ultimate success when cases are appropriately selected for staging [8]. Likewise, Kaplan–Meier survival analysis did not reveal differences in time-to-decannulation, suggesting that stage selection did not disadvantage either group in long-term airway patency. The decannulation trajectory is illustrated in Fig. 4.

The apparent discrepancy between shorter median time to decannulation in the SS group and non significant Kaplan–Meier (Fig. 3) analysis suggests that the difference is primarily driven by early postoperative decannulation events rather than sustained divergence in long-term airway outcomes.To account for variable follow-up duration, we performed a sensitivity analysis limited to patients with at least 6 months of follow-up. Decannulation rates remained high and did not differ significantly between staging strategies, suggesting that early differences in median time to decannulation do not reflect divergence in ultimate long-term airway outcomes.

### Predictors of decannulation timing and success

Our findings demonstrate that stenosis severity appears strongly associated with decannulation outcomes. Both categorical and time-to-event analyses consistently identified Cotton–Myer grade IV stenosis as a significant predictor of delayed decannulation. This observation aligns with prior reports demonstrating that higher-grade stenosis reflects more extensive airway scarring, reduced airway diameter, and increased reconstruction complexity, all of which may prolong airway stabilization and delay decannulation [8, 25].

Importantly, the number of postoperative endoscopic airway procedures was strongly associated with delayed decannulation. This likely reflects underlying airway complexity rather than procedural harm, as patients requiring more frequent surveillance and intervention typically have more severe or dynamic airway pathology. Similar associations between procedural burden and delayed airway recovery have been described in revision airway reconstruction cohorts and patients with multilevel stenosis [27].

In contrast, demographic factors such as age and sex, as well as operative variables including stent use, were not independently associated with time to decannulation. These findings suggest that intrinsic airway disease severity may be more associated with recovery trajectory than operative technique alone determinant of recovery trajectory.

### Complications, infection, and microbiology

Postoperative infection occurred exclusively in stented patients and was significantly associated with stent use on univariate analysis. This association likely reflects underlying disease complexity rather than a direct causal effect of the stent itself, as patients requiring stents typically had more extensive stenosis or less stable airway reconstructions. In addition, shorter duration of tracheostomy prior to reconstruction was associated with increased infection risk. While this finding may appear counterintuitive, it may reflect more aggressive surgical intervention in patients with rapidly progressive disease or complex anatomy, rather than a protective effect of prolonged tracheostomy.

These findings are consistent with prior literature demonstrating that airway stent use and severe airway pathology are associated with increased risk of infection, likely mediated by impaired mucociliary clearance, biofilm formation, and bacterial colonization [21–23].

Microbiologically, our cohort documented high rates of bacterial colonization (70.5%), dominated by *Pseudomonas aeruginosa*, paralleling colonization patterns reported in stented airways and long-term tracheostomies [26].

### Sedation withdrawal

Sedation withdrawal syndrome, also referred to in the literature as iatrogenic withdrawal syndrome (IWS), is a clinical phenomenon that may occur in children exposed to prolonged sedative and analgesic therapy, most commonly opioids and benzodiazepines. It arises when tolerance and physical dependence develop during continuous infusion, with withdrawal symptoms emerging during rapid tapering or abrupt discontinuation. Clinically affected children may exhibit a constellation of autonomic, neurologic, and gastrointestinal manifestations, including agitation, tremors, hypertension, tachycardia, irritability, and feeding intolerance [25].

In our cohort, DS reconstruction was associated with a lower incidence of withdrawal syndrome, potentially reflecting differences in postoperative airway management. Tracheostomized DS patients typically avoid prolonged postoperative intubation and deep sedation, whereas SS reconstruction often necessitates several days of sedation to protect the airway and graft and to minimize the risk of inadvertent extubation.

To our knowledge, the association between surgical staging strategy, graft-related considerations, and the risk of sedation withdrawal has not been previously described in the pediatric airway reconstruction literature and adds to the understanding of perioperative morbidity in this population.

The observed difference in withdrawal syndrome between staging strategies likely reflects differences in postoperative airway management rather than intrinsic patient physiology. SS reconstruction requires planned prolonged intubation and continuous sedative infusion to protect the airway graft, whereas DS reconstruction maintains tracheostomy patency and typically avoids extended mechanical ventilation. Accordingly, increased withdrawal risk in the SS group should be interpreted as a consequence of management strategy rather than a biologic effect of staging itself.

### Follow-up and airway surveillance

**Fig. 5:**
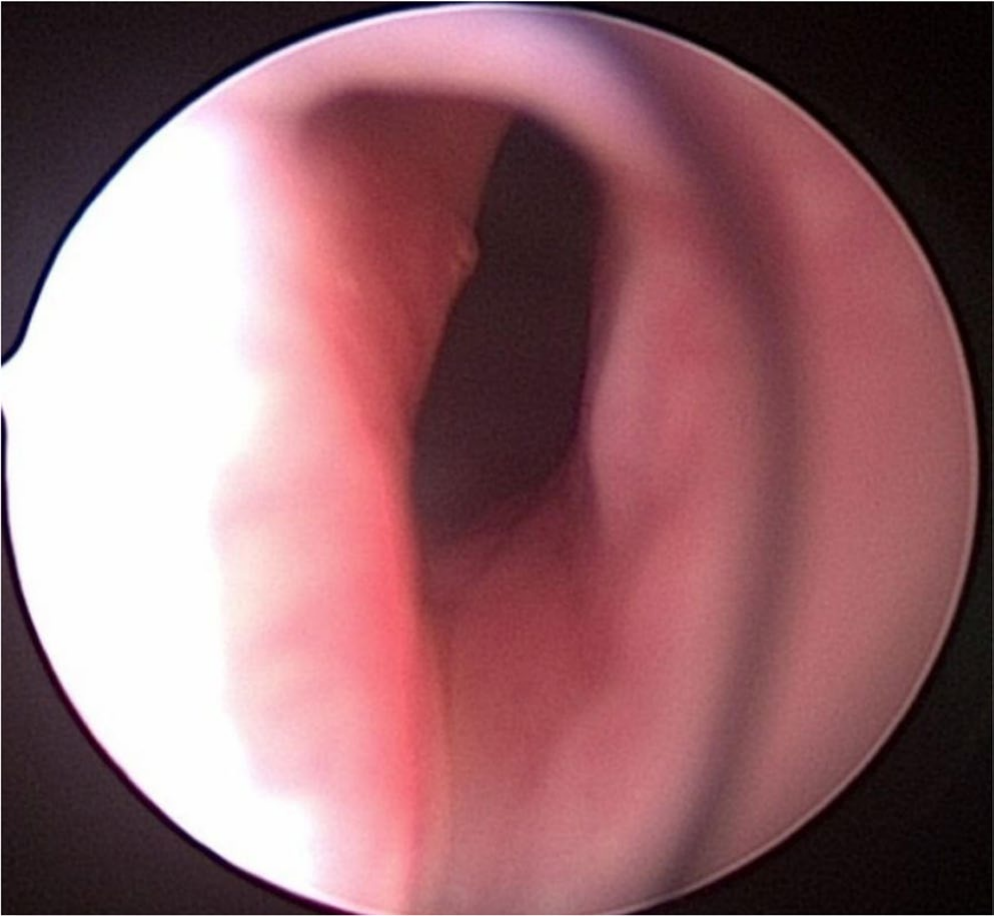
Direct laryngoscopy 1 month postoperative

Our cohort had a median follow-up of approximately 5 months (IQR 3–29 months), during which 16% remained tracheostomy dependent. Successfully decannulated patients underwent a median of 2.2 postoperative DLs before safe decannulation, an example is shown in Fig. 5, in line with typical recommendations for serial micro laryngoscopy to monitor graft stability and assess supraglottic/laryngeal healing [25].

This study has several important limitations. First, its retrospective, single-center design introduces inherent selection and information bias and may limit generalizability. Surgical staging, operative approach, and stent use were determined by surgeon judgment based on airway severity, anatomical complexity, and patient comorbidities rather than randomized allocation, introducing substantial selection bias in comparisons between single-stage and double-stage reconstruction. Second, operative techniques and postoperative management protocols evolved over the 10-year study period, including a marked reduction in stent utilization and variation in sedation practices, resulting in temporal and management heterogeneity. Sedation duration was not uniformly captured, limiting precise assessment of exposure-response relationships for withdrawal syndrome. Third, the modest sample size restricts statistical power, particularly for multivariable modeling of relatively infrequent outcomes such as postoperative infection. Finally, variable follow-up duration, with many patients followed for less than six months, limits definitive conclusions regarding long-term airway durability. Although sensitivity analysis demonstrated similar decannulation rates among patients with adequate follow-up, longer standardized follow-up would provide more robust assessment of sustained airway success. Accordingly, observed associations should be interpreted as descriptive rather than causal and warrant validation in larger prospective cohorts.

In pediatric laryngotracheal reconstruction, decannulation alone does not fully capture the complexity of patient outcomes. Our findings suggest that surgical staging, stent use, and perioperative sedation strategies were associated with differences in postoperative morbiditys. While SS and DS approaches achieve comparable decannulation trajectories when appropriately selected, their complication profiles differ, particularly regarding infection and withdrawal syndrome. These observations highlight the need for individualized surgical planning and underscore the importance of evaluating broader clinical endpoints beyond airway patency. Future prospective, multicenter studies are warranted to refine staging algorithms and optimize perioperative management.

## Conclusion

Pediatric open airway reconstruction outcomes extend beyond decannulation and include perioperative morbidity. In this cohort, postoperative outcomes varied according to staging strategy, stent utilization and duration of preoperative tracheostomy. The marked decline in stent use over time reflects an evolution in practice that may mitigate infectious complications without compromising decannulation success. Together, these findings suggest that success in pediatric airway surgery should be evaluated beyond decannulation alone. Pediatric airway surgery should be addressed as a balance between airway patency and minimization of treatment-related morbidity through individualized, data-driven surgical planning.

## Supplementary Information

Below is the link to the electronic supplementary material.

Supplementary Material 1
