# Supplementary material for: A comprehensive 10-year review of decannulation and perioperative complications in pediatric open airway reconstruction
Source: Eur Arch Otorhinolaryngol. 2026 Apr 23;283(6):3991–4001. doi: 10.1007/s00405-026-10154-7 (PMC13249641; doi:10.1007/s00405-026-10154-7)
Supplement: Supplementary file 1 — Supplementary Material 1 [file 405_2026_10154_MOESM1_ESM.docx]

February 24^th^, 2026

Manuel Bernal-Sprekelsen MD, PhD, Prof. Andreas Knopf

Editor-in-Chief, *European Archives of Oto-Rhino-Laryngology*

Dear Dr. Bernal-Sprekelsen and Prof. Andreas Knopf,

Attached please find our manuscript entitled **“ A Comprehensive 10-Year Review of Decannulation and Perioperative Complications in Pediatric Open Airway Reconstruction”** which we submit for consideration for publication *European Archives of Oto-Rhino-Laryngology .*

In this retrospective cohort study of pediatric open airway reconstruction, we examine perioperative morbidity, microbiologic findings, and decannulation outcomes, emphasizing clinically meaningful endpoints beyond airway patency. Our results show that while single- and double-stage reconstructions achieve comparable decannulation outcomes when appropriately selected, differences in stent use, infection rates, and sedation withdrawal highlight modifiable perioperative factors that influence recovery.

We believe this work provides practical insights into contemporary pediatric airway management and aligns well with the journal’s mission to advance evidence-based otolaryngologic care.

This manuscript has not been published previously and is not under consideration elsewhere. All authors have approved the manuscript and declare no conflicts of interest.

Hen Chaushu, MD

Tel Aviv Sourasky Medical Center

henchaushu@gmail.com
